# Synchronous occurrence of Waldenström macroglobulinemia and HER2-positive gastric adenocarcinoma with gastrointestinal stromal tumor: a rare case report

**DOI:** 10.3389/fonc.2025.1554206

**Published:** 2025-05-16

**Authors:** Yingming Jin, Zhilong Shi, Kesang Li, Tianjiao Liu, Zhi Fang

**Affiliations:** Department of Hematology and Oncology, Ningbo No.2 Hospital, Ningbo, China

**Keywords:** Waldenström macroglobulinemia, gastric adenocarcinoma, gastrointestinal stromal tumor (GIST), human epidermal growth factor receptor-2 (HER-2), synchronous occurrence

## Abstract

There are very few reports of gastric cancer (GC) and gastrointestinal stromal tumor (GIST) occurring at the same time in the literature. In particular, the collision of GIST and human epidermal growth factor receptor-2 (HER-2)-positive gastric carcinoma in a patient with Waldenström macroglobulinemia (WM) has never been reported. We report the case of an 80-year-old male who initially presented with dizziness with fatigue for more than 1 month. He underwent total gastrectomy. Pathology revealed GC (moderately to poorly differentiated tubular adenocarcinoma), GIST (low-risk category) and low-grade small B-cell lymphoma with plasmacytoid differentiation. Furthermore, the diagnosis of WM was made after cytomorphologic and immunohistochemical analysis of the patient’s bone marrow revealed the presence of lymphoplasma cells along with the *MYD88 ^L265P^* mutation and an increased level of serum monoclonal immunoglobulin M (IgM). To our knowledge, this is the first case of such an association where WM occurred with concomitant HER2-positive gastric adenocarcinoma and gastric GIST. A review of the literature concerning the incredibly uncommon simultaneous triple incidence of malignant tumors with distinct histogenesis is presented below.

## Introduction

GC is a prevalent malignant tumor originating from epithelial tissue. Adenocarcinoma is the most common type of GC. Globally, the reported HER-2-positive rate in GC ranges from 7.3% to 20.2% (1). GIST, accounting for 1%~2% of gastrointestinal tumors, is the most common mesenchymal tumors and are most commonly observed in the stomach (50~60%) (2). However, the prevalence of both GC and GIST is between 0.29% and 0.53% (3). WM is an uncommon type of B-cell lymphoma in which the IgM protein is the hallmark of the disease. There are few reports of synchronous WM and second cancers. However, there has never been a report of GIST and HER-2-positive stomach cancer colliding in a WM patient. Herein, we present an extremely unwonted case of these three lesions.

## Case presentation

**Figure 1:**
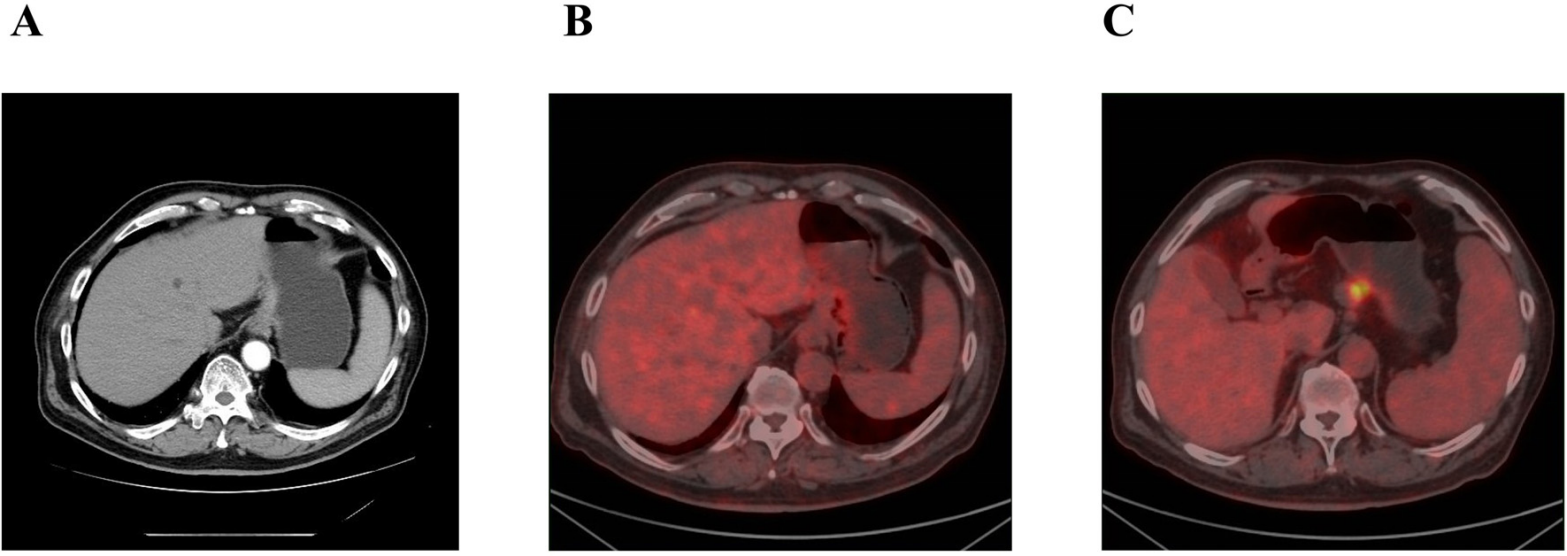
**(A)** Abdominal enhanved CT: uneven thickening augmentation of the gastric fundus. **(B, C)** PET-CT: **(B)** thickening of the gastric wall. **(C)** lymph node with increased metabolism of FDG.

An 80-year-old male was admitted to the Department of Gastrointestinal Surgery at our hospital on 8 September 2023 due to dizziness accompanied by fatigue and a poor appetite for more than a month. He had a history of cerebral infarction, hypertension, and prostatic hyperplasia. One month before hospitalization, the patient visited the local hospital for gastroscopy examination, and gastroscopy revealed space-occupying lesions in the fundus and body of the stomach. Pathologic examination revealed poorly differentiated adenocarcinoma. He was referred to our hospital for further treatment. Additionally, uneven thickening and augmentation of the gastric fundus wall were identified via abdominal contrast-enhanced computed tomography (**Figure 1A**). Positron emission tomography-computed tomography (PET-CT) confirmed thickening of the gastric wall on the side of the gastric cardia and lesser curvature with increased metabolism of FDG and multiple lymph node metastases beside the lesser curvature of the stomach, hepatogastric space, portal of the liver, retroperitoneum, superior diaphragm, and lower esophagus (**Figures 1B, C**).

**Figure 2:**
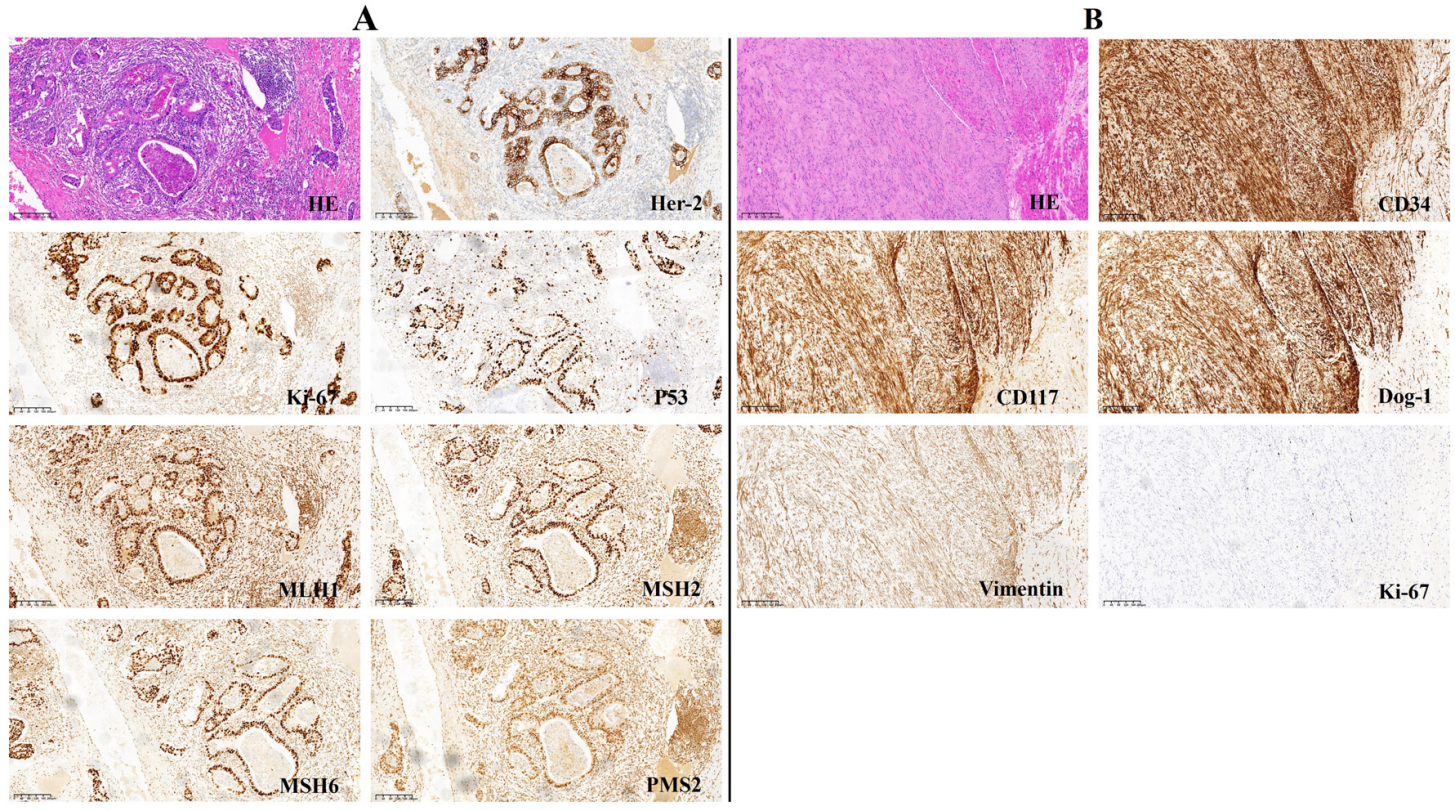
Immunohistochemistry analysis (magnification, x 100). **(A)** gastric adenocarcinoma. **(B)** GIST.

**Figure 3:**
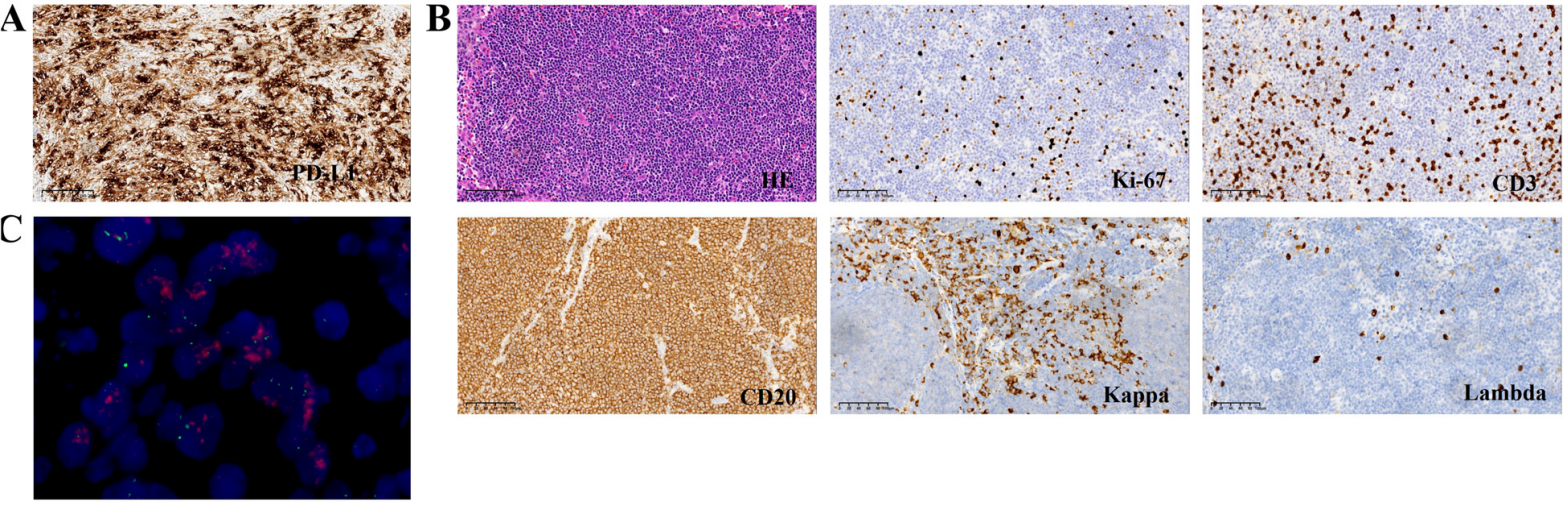
**(A)** Immunochemistry analysis showing expression of PD-L1. **(B)** Immunochemistry analysis (magnification, x 200) showing CD20+/CD3-/Kappa+/Lambda-/Ki-67 low phenotype of the No.8 Lymph node. **(C)** FISH showing HER-2 positive.

He underwent laparoscopy-assisted total gastrectomy with Roux-en-Y esophagojejunostomy and D2 lymph node dissection on 9 September 2023. The operation was successful, with uneventful outcomes. The postoperative pathological examination of the stomach revealed a moderately to poorly differentiated tubular adenocarcinoma that had not spread to the visceral peritoneum but had infiltrated the connective tissue of the subserous membrane. The tumor was located in the lesser curvature of the body of the stomach and measured 40mm*33mm*12mm in size. Ten of the 25 resected lymph nodes were found to contain metastases, and all margins were negative (pT3N2aM0, Stage IIIB). Immunohistochemical findings included CK7 (-), CEA (focal +), CK20 (focal +), CDX-2 (+), E-cadherin (+), Her-2 (2+), Ki-67 (+80%), P53 (overexpress), and EBER (-). Mismatch repair (MMR) protein expression was intact (**Figure 2A**). Programmed death-ligand 1 (PD-L1) combined positive score (CPS) 50 (CPS < 1 is negative, and CPS≥1 is positive) (**Figure 3A**). The histopathology of the gastric cardia revealed GIST characteristics, including spindle-shaped cells, a mitotic ratio of 1/50HPF, neither substantial atypia nor visible necrosis. Immunostaining for CD34, vimentin (+) and c-kit was positive, whereas S-100, SMA and desmin were negative. GISTs were classified as very low risk according to the NIH2008 modified version (**Figure 2B**; **Supplementary Table 1A**).

Furthermore, No.8 Lymph node analysis revealed low-grade small B-cell lymphoma with plasmacytoid differentiation. Immunohistochemistry revealed the following: kappa (+), lambda (-), PAX-5 (+), Ki-67 (+10%), Bcl-2 (+), Bcl-6 (-), CyclinD1 (-), SOX-11 (-), CD3 (-), CD5 (-), CD10 (-), CD20 (+), CD21 (+), CD23 (+), CD43 (-), and CD79a (+)(**Figure 3B**; **Supplementary Table 1A**).

**Figure 4:**
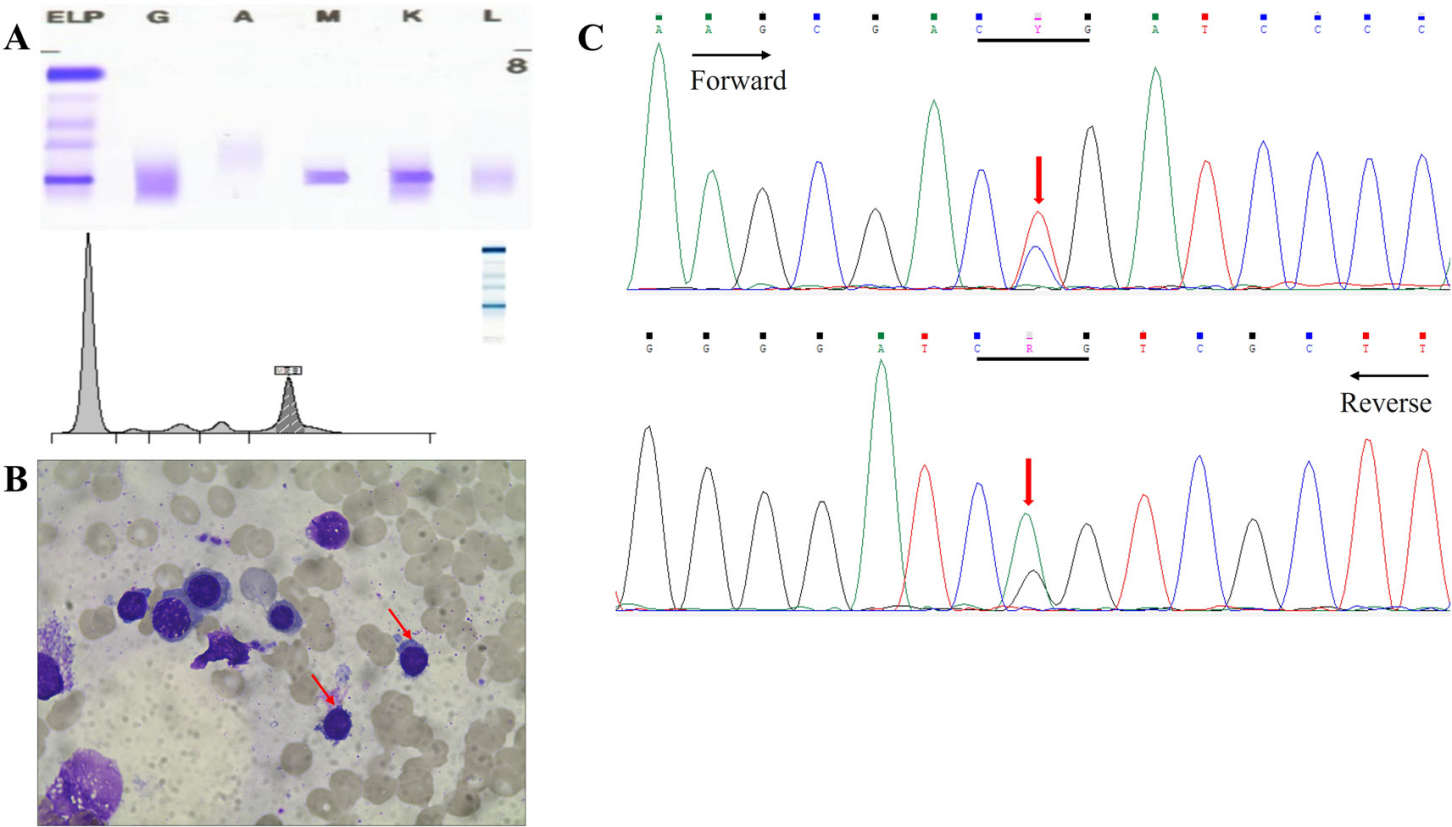
**(A)** Serum electrophoresis and immunofixation electrophoresis revealed IgM κ. **(B)** Bone marrow puncture showed lymphoplasma cells. **(C)** Perform *MYD88^L265P^* mutational analysis on bone marrow by Sanger sequencing. Y:C/T R:A/G.

He was subsequently hospitalized in our wards in November 2023 for further diagnosis and treatment. A laboratory test revealed mild anemia (hemoglobin of 94 g/L) together with low white blood cell (2.9×10^9^/L) and platelet (111×10^9^/L) counts. Biochemical tests revealed hyperphosphatemia (5.11 mmol/L), normal lactate dehydrogenase (120 U/L), and increased IgM (24.3 g/L). Serum beta-2-microglobulin (3.49 mg/L) was also above normal. The monoclonal immunoglobulin was identified as IgM-κ by serum immunofixation electrophoresis analysis, and immunoglobulin quantification was carried out (**Figure 4A**). Subsequently, bone marrow aspiration and biopsy were performed. The patient’s bone marrow smears revealed a small number of lymphoplasma cells (**Figure 4B**). Immunotyping revealed that CD45+CD19+ cells accounted for 11.4% of nuclear cells and expressed HLA-DR, CD19, CD22 and sKappa without CD5, CD10, CD11c, CD23, CD38, CD103, CD200 and Lambda. Cytogenetics revealed a normal karyotype. A mutation in *MYD88 L265P*, was detected in the patient via molecular biological testing (**Figure 4C**).

BM aspiration confirmed the diagnosis of a mature B-cell neoplasm. No osteolytic bone lesions were found in association with multiple myeloma. Calcium and creatinine levels were normal. Eventually, WM was diagnosed. The patient had a high risk score according to the International Prognostic System for WM. Owing to his advanced age and poor performance, the patient and his family declined chemotherapy. Zanubrutinib (160 mg orally twice a day) and supportive care were given to the patient because of exhaustion, anemia, and dizziness. Four months later, the patient was hospitalized in our department due to dysphagia, vomiting after eating, and defecation difficulties. The tumor markers were elevated (**Supplementary Table 1B**), and gastroscopy revealed extensive hyperemic erosion of the esophageal mucosa with bleeding. We considered the possibility of gastric cancer recurrence, and zanubrutinib was suspended. HER-2 was positive on the basis of the combined interpretation of the results of the IHC assays (2+) and FISH (+) (**Figure 3C**). Given that his PD-L1 CPS was 50, he was administered immune checkpoint inhibitors plus anti-HER-2 treatment on April 4, 2024. Unfortunately, the patient refused further immunotherapy and targeted therapy because of poor performance status and was treated with traditional Chinese medicine without any outpatient follow-up for personal reasons.

## Discussion

GCs are currently the fifth most common cancer worldwide, with approximately 75% of cases occurring in Asia, whereas GISTs represent the most common mesenchymal neoplasms of the digestive tract, with an incidence of 50~60% in the stomach (2, 4). However, the coexistence of GCs and GISTs is uncommon and is often detected incidentally during surgery, as in our case. It has been reported that the synchronous occurrence of GC and GIST is 0.29%~0.53% (3). Globally, there are very few case reports on GC with GIST. Notably, in our patient, HER-2 was positive on the basis of the combined interpretation of the IHC (2+) and FISH (+) results (5). Surprisingly, to the best of our knowledge, no cases of HER-2-positive GC or GIST have been reported thus far, and the prognostic role of HER-2 overexpression in GC is still controversial (6, 7). Typically, GISTs have low malignant potential, whereas GCs are usually advanced. However, the etiology of GC occurring simultaneously with GIST is still unclear. The co-occurrence of GC and GIST is thought by some researchers to be accidental, but other research has suggested that it may be linked to common carcinogens such as nitrite and *Helicobacter pylori* (8–10). However, there is currently no evidence linking GIST to *H. pylori* infection or clue of *H. pylori* infection in the patient. Gene mutations may cause two neighboring tissues to interact, disrupting the regulation of mesothelial and epithelial cell development and resulting in distinct cancers in two tissues of the same organ.

WM is a type of lymphoplasmacytic lymphoma with IgM monoclonal protein. It accounts for approximately 1~2% of hematologic malignancies. More than 90% of WM patients have the *MYD88 ^L265P^* mutation (11, 12). The coexistence of gastric adenocarcinoma with lymphoma has been documented in a few cases. Most patients have GC diagnosed alongside non-Hodgkin lymphoma, including diffuse large B-cell lymphoma (DLBCL) and mucosa-associated lymphoid tissue (MALT) lymphoma (13–16). Feng et al. (16) reported that stomach cancer and precancerous abnormalities were present in 5.1% and 14.6% of patients with primary gastric lymphoma, respectively. However, according to our literature review, we rarely observe two cases of synchronous triple occurrence: adenocarcinoma, lymphoma, and GIST in the stomach. One patient had MALT lymphoma, and the other had DLBCL (10, 17). There are few reports of synchronous WM and second cancers. A total of 225 (24%) of the 924 patients with WM developed secondary malignancy (SM), although more than 60% of the cases were seen before the diagnosis of WM was made. The most frequent secondary cancers in that study were those of the breast, skin, hematologic, melanoma, lung, and thyroid (18). A retrospective analysis of 230 consecutive WM patients was conducted by Varettoni et al. (19). At the time of the WM diagnosis, 17 patients (7%) had a history of one (n = 15) or two (n = 2) solid malignancies. In another study, patients who were exposed to nucleoside analogs seemed to have a greater risk of developing MDS or AML (20). In a follow-up study, patients with WM who received treatment had a fivefold increased chance of developing secondary cancers (21). According to an analysis of the Surveillance, Epidemiology, and End Results (SEER) database (1992–2011), 681 SMs (14.56%) were found in 4676 WM patients, and the SM risk was 49% greater in WM patients than in the general population. At 5 and 10 years, the cumulative incidence of SMs was 10% and 16%, respectively (22). Our search of the literature revealed that there were only 3 cases of synchronous macroglobulinemia with stomach cancer (23–25). Coincidentally, 2 patients were reported in Japanese articles without available abstracts. Given the high incidence of stomach cancer in East Asian nations, the co-occurrence of macroglobulinemia and gastric cancer appears to be random from a clinical standpoint. The etiology of WM with second cancer is unknown. The occurrence of second tumors in WM patients may be associated with immune defects such as B-cell dysfunction, low gamma globulin levels and T-cell subgroup dysfunction, as well as treatment and genetic susceptibility (20).

The lack of concurrent treatment approaches makes managing multiple synchronous primary tumors challenging. MDT (multidisciplinary team) discussion is considered an essential component of care for patients with multiple malignant tumors. The therapeutic approach should be chosen on the basis of the experience of the physician in the absence of recommendations for the management of such disorders. Personalized treatment plans are suggested on the basis of the patient’s condition. For patients with HER2-positive disease, the recommended first-line regimen is trastuzumab (anti-HER2) in combination with platinum and fluoropyrimidine-based chemotherapy. However, the goal of individualized therapy is to avoid unnecessary therapeutic interventions for patients who are unlikely to respond to therapy. In our case, the frail elderly patient had poor nutritional status after total gastrectomy and was bed-ridden. Unfortunately, the patient refused further immunotherapy and targeted therapy and was treated with traditional Chinese medicine without any outpatient follow-up for personal reasons.

The limitations of our case are as follows: the patient refused next-generation sequencing (NGS) because of his advanced age and poor physical status. Hence, the genetic mutation was unknown. Additionally, owing to advanced age and poor tolerance, treatment cannot benefit from immunotherapy or targeted therapy.

This case is unique, as it demonstrates the rare coexistence of WM, gastric adenocarcinoma, and gastric GIST. Considering that enlarged lymph nodes often occur in solid tumors, clinicians and pathologists tend to overlook the possibility of secondary tumors such as lymphoma. Therefore, such cases highlight the importance of postoperative pathology in lymph node dissection. Additional cases, therefore, need to be investigated to further clarify the key diagnostic and therapeutic characteristics of synchronous neoplasms. Therefore, more cases need to be investigated to better understand the essential diagnostic and treatment features of synchronous malignancies.

## Data Availability

The datasets presented in this study can be found in online repositories. The names of the repository/repositories and accession number(s) can be found in the article/**Supplementary Material**.
