# Supplementary material for: Synchronous occurrence of Waldenström macroglobulinemia and HER2-positive gastric adenocarcinoma with gastrointestinal stromal tumor: a rare case report
Source: Front Oncol. 2025 May 16;15:1554206. doi: 10.3389/fonc.2025.1554206 (PMC12122335; doi:10.3389/fonc.2025.1554206)
Supplement: Supplementary file 1 [file DataSheet1.pdf]

HER2 Gene Detection Probe Manual

【Probe Description】 Dual color probes based on Fluorescence in situ Hybridization.

Name	Pack size	Amount	Component
	5 tests/kit		
HER2 Gene Probe	50 μl/vial	1 vial	GSP HER2 (red)、CSP 17(green);<br>formamide、SSC、dextran sulfate

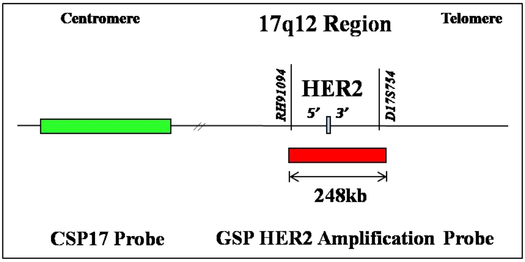

【Storage instructions】 Stored at -20±5℃ and protected from light. The validity is set for 18 months.

【Equipment and Accessories】 Fluorescence microscope equipped with recommended filters.

DAPI (364/454)、Green (495/521) and Orange (551/575).

【Signal Enumeration】

Determine and record the number of GSP HER2 and CSP17 counts in 20 nuclei for at least 2 invasive carcinoma area. For each nucleus, count the number of GSP HER2 and CSP17 signals. Add all GSP HER2 signals, and then to calculate the ratio of GSP HER2/CSP 17.

a) If the HER2/CSP17 ratio is <2.0 and the average HER2 gene copy number is <4.0 per tumor cell, HER2 gene amplification was not observed.

b) If the HER2/CSP17 ratio is <2.0 ,but the average HER2 gene copy number is ≥6.0 per tumor cell, HER2 gene amplification was observed.

c) If the GSP HER2 / CSP17 ratio is ≥2.0 , HER2 gene amplification was observed.

d) If GSP HER2 signals clusters and/or multiple dots, HER2 gene amplification was observed.

e) If the HER2/CEP17 ratio is <2.0 and an average HER2 gene copy number is <6.0,but ≥ 4.0 per tumor cell, the HER2 status was regarded as equivocal. Count an additional 20 nuclei and recalculate the ratio based on the total of 40 nuclei.

【Manufacturer information】

Manufacturer: Guang Zhou LBP Medicine Science & Technology Co., Ltd.

Distributor: JOKOH CO.,LTD.

Address: 154 Nishikata Kikugawa-shi Shizuoka TEL: 0537-88-7318

Cat. F.01359

HER2 Gene Detection Probe

HER2 基因检测探针

Pack size 5 tests/kit

Storage -20±5℃ (protected from light)

For research use only, not for clinical use.

Please refer to the instruction for other details.

Manufacturer: Guang Zhou LBP Medicine Science & Technology Co.,Ltd.

Distributor: JOKOH CO.,LTD.

Address: 154 Nishikata Kikugawa-shi Shizuoka

Tel: 0537-88-7318

Batch lot:

Production date:

Expiry Date:

LBP 安必平®
