# Supplementary material for: Synchronous occurrence of Waldenström macroglobulinemia and HER2-positive gastric adenocarcinoma with gastrointestinal stromal tumor: a rare case report
Source: Front Oncol. 2025 May 16;15:1554206. doi: 10.3389/fonc.2025.1554206 (PMC12122335; doi:10.3389/fonc.2025.1554206)

Supplementary 1

1. **Clone names for immunohistochemistry antibodies**

| Antibody | Clone name | Antibody | Clone name | Antibody | Clone name | Antibody | Clone  name |
| --- | --- | --- | --- | --- | --- | --- | --- |
| Bcl-2 | SP66 | CD43 | L60 | Her-2 | 4B5 | PD-L1 | 22C3 |
| Bcl-6 | LN22 | CD79a | SP18C | Kappa | L1C1 | PMS2 | OTI4B2 |
| CD3 | LN10 | CD117 | 2E4 | Ki-67 | UMAB107 | S-100 | 15E2E2+4C4.9 |
| CD5 | 4C7 | CDX-2 | EP25 | Lambda | BP6133 | SMA | CK1 |
| CD10 | BP6059 | CEA | COL-1 | MLH1 | OTI4H4 | SOX-11 | BP6070 |
| CD20 | L26 | CK7 | UMAB161 | MSH2 | BPM6143 | Vimentin | UMAB159 |
| CD21 | EP64 | CK20 | EP23 | MSH6 | EP49 |  |  |
| CD23 | UMAB101 | CyclinD1 | SP4-R | P53 | DO-7 |  |  |
| CD34 | 10C9 | E-cadherin | EP6 | PAX-5 | SP34 |  |  |

1. **The elevated tumor markers**


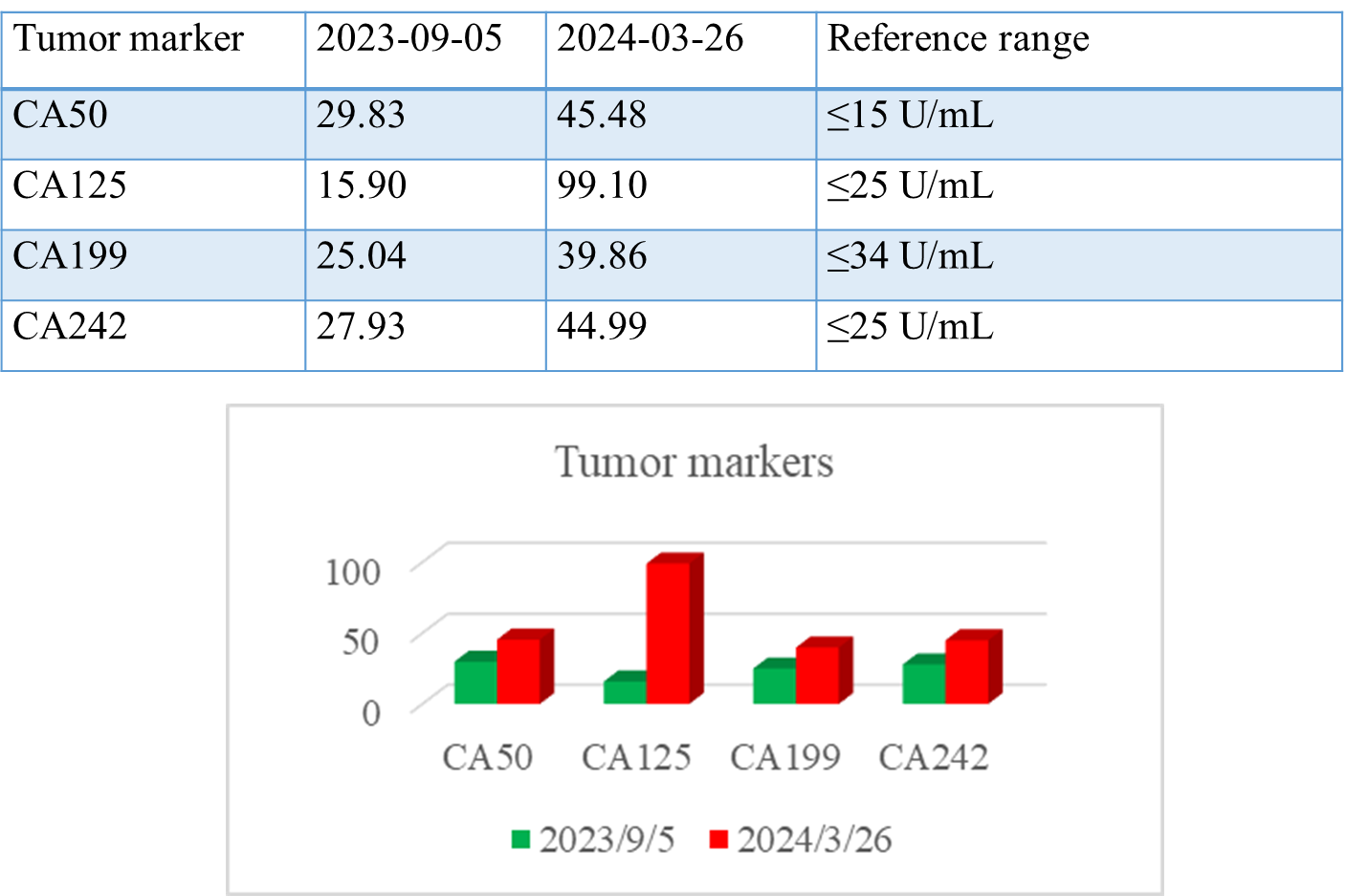

Supplement: Supplementary file 2 [file Table1.docx]
